# Utilization of natural products in diverse pathogeneses of diseases associated with single or double DNA strand damage repair

**DOI:** 10.1186/s13020-025-01089-y

**Published:** 2025-04-07

**Authors:** Jiali Liang, Wanqing Liu, Tong Zhang, Dean Guo, Jiyu Gong, Zizhao Yang

**Affiliations:** 1 School of Pharmacy, Shanghai University of Traditional Chinese Medicine, Shanghai, 201203 China; 2 School of Pharmaceutical Sciences, Changchun University of Chinese Medicine, Changchun, 130117 China; 3 Zhongshan Institute for Drug Discovery, Shanghai Institute of Materia Medica, Chinese Academy of Sciences, Zhongshan, 528400 China; 4 Center for Laboratory Animal Service and Experiments, Seventh People’s Hospital of Shanghai University of Traditional Chinese Medicine, Shanghai, 200137 China

**Keywords:** Single or double strands DNA damage, Enzymes in DNA repair, Natural products, Pathogeneses, Clinical treatment strategy of DNA damage involved maladies

## Abstract

The appearance of DNA damage often involves the participation of related enzymes, which can affect the onset and development of various diseases. Several natural active compounds have been found to efficiently adjust the activity of crucial enzymes associated with single or double-strand DNA damage, thus demonstrating their promise in treating diseases. This paper provides an in-depth examination and summary of these modulation mechanisms, leading to a thorough review of the subject. The connection between natural active compounds and disease development is explored through an analysis of the structural characteristics of these compounds. By reviewing how different scholarly sources describe identical structures using varied terminology, this study also delves into their effects on enzyme regulation. This review offers an in-depth examination of how natural active compounds can potentially be used therapeutically to influence key enzyme activities or expression levels, which in turn can affect the process of DNA damage repair (DDR). These natural compounds have been shown to not only reduce the occurrence of DNA damage but also boost the efficiency of repair processes, presenting new therapeutic opportunities for conditions such as cancer and other disease pathologies. Future research should focus on clarifying the exact mechanisms of these compounds to maximize their clinical utility and support the creation of novel approaches for disease prevention and treatment.

**Graphical abstract:**

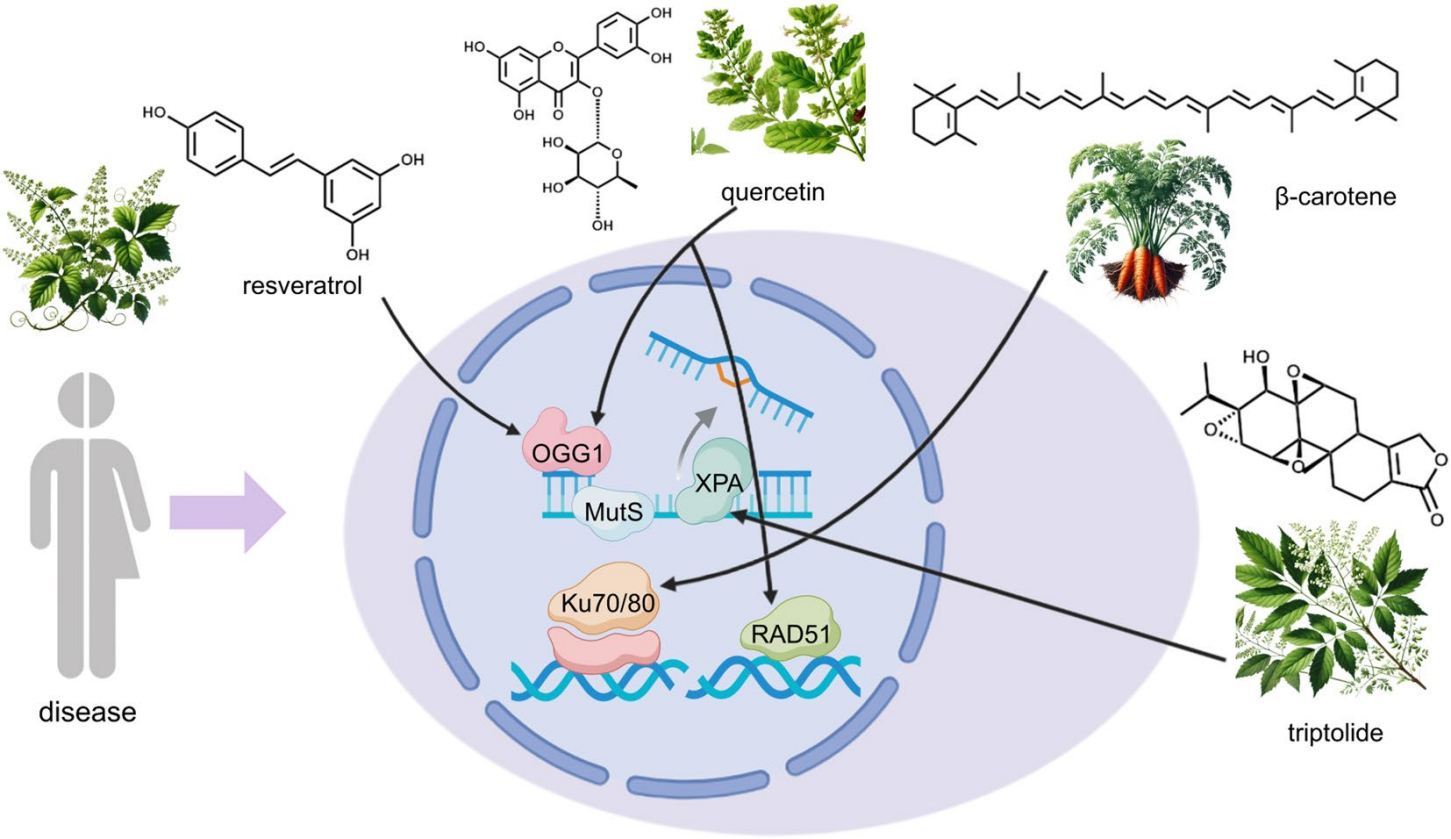

## Highlights

1. The lactone constituent triptolide effectively modulates key enzymes involved in SSBs like BER, NER or DSBs like NHEJ or HRR, thereby impacting the initiation and progression of tumorigenic diseases.
2. The flavonoid compound quercetin not only regulates OGG1 in BER, but also modulates RAD51 and BRCA1 in HRR, thereby impacting the developments and deteriorations of multiple maladies.
3. The alkaloid berberine, a natural compound, has shown effectiveness in treating TNBC and NSCLC. It achieves this not only by regulating XRCC1 in the BER pathway but also by influencing the NER process.

## Introduction

DNA damage represents a substantial risk to genomic stability, necessitating the activation of a range of cellular mechanisms known collectively as DNA damage repair (DDR). These processes work together to effectively address and minimize the damage, ensuring the preservation of cellular functions [1, 2]. DNA damage primarily manifests in two forms: single-strand breaks (SSBs) and double-strand breaks (DSBs). These two types of damage represent the major classifications of DNA lesions, each characterized by distinct features and implications for cellular function and genomic stability. The DDR system operates through five critical repair pathways: base excision repair (BER) for addressing small base lesions, nucleotide excision repair (NER) for removing bulky adducts, mismatch repair (MMR) for correcting replication errors, non-homologous end joining (NHEJ) for repairing DSBs, and homologous recombination repair (HRR) for resolving complex DNA damage. Each pathway specifically targets distinct types of DNA damage [3–5]. Moreover, specific forms of DNA damage employ dedicated tolerance pathways, such as translesion synthesis (TLS) mechanisms [6, 7]. Although cells exhibit varied responses to different types of DNA damage, including SSBs, DSBs and base modifications, an underlying and consistent set of mechanisms oversees the repair and handling of such damages [1, 8]. The repair of DNA damage can be directly facilitated by key enzymes in the pathway [9]. Most damage, however, is rectified through a sequential cascade of catalytic events facilitated by multiple key enzymes.

SSBs are the most frequent form of DNA lesions, characterized by interruptions in the DNA double helix. These lesions are frequently associated with nucleotide depletion and damage to the 5ʹ and 3ʹ termini at the break site [10]. Delayed repair of SSBs can severely compromise genomic stability and threaten cell viability. This delay not only disrupts the integrity and functionality of genetic material but also impairs cellular proliferation and the maintenance of normal physiological processes. Without prompt intervention to repair these breaks, the stability that underpins the entire cellular and genetic machinery is placed at significant risk, potentially leading to adverse consequences for cellular survival [11]. The cells have developed a rapid repair mechanism known as single-strand break repair (SSBR) in response to this, ensuring efficient DNA damage restoration [12]. The SSBR process is often categorized as a specialized variant of BER because it involves key enzymes primarily linked with the BER pathway. Importantly, poly (ADP-ribose) polymerase 1 (PARP1) and X-ray repair cross-complementing protein 1 (XRCC1) are two critical enzymes that significantly contribute to orchestrating SSBR. Their collaborative activities are essential for ensuring efficient and accurate repair of SSBs in DNA, highlighting the close association between SSBR and the overall BER mechanism [13, 14]. Most SSBs are corrected via a highly efficient and universal SSBR pathway. This pathway can be broken down into four key phases. In the first phase, the detection of SSBs triggers the activation of the repair process. The second stage entails processing the DNA ends, during which obstructive components at both the 5ʹ and 3ʹ ends are removed. Following the identification of SSBs, the DNA gap is filled during the DNA gap filling stage. Subsequently, in the DNA ligation stage, the repaired segments are covalently joined. To summarize, the effective SSBR mechanism that addresses the majority of SSBs involves four essential stages: recognition of SSBs, processing of DNA ends, filling of DNA gaps, and ligation of DNA [15].

The induction of DSBs can result in severe genomic damage, leading to chromosomal rearrangements that ultimately trigger cell death. A wide array of human diseases, including developmental abnormalities and various forms of cancer, originate from this mechanism [16–19]. The two primary mechanisms of DSBs that predominate in mammals are NHEJ and HRR [20]. The onset of the NHEJ mechanism starts when a complex formed by the proteins Ku70 and Ku80 (referred to jointly as Ku) identifies DNA DSBs [21]. The catalytic subunit of DNA-dependent protein kinase (DNA-PKcs) exhibits strong interaction with DNA ends, a process that is significantly amplified when Ku attaches to these areas [22, 23]. The nuclease Artemis is tightly associated with DNA-PKcs and is likely recruited in concert with this kind of kinase. Nucleotide incorporation is carried out by polymerases belonging to the Pol X family, notably Pol μ and Pol λ. The DNA ligase IV (LIG4) complex, comprising XRCC4, XLF, and possibly PAXX, is crucial for sealing DSBs [24, 25]. Unlike NHEJ, which is active in repairing DSBs across all phases of the cell cycle, HRR primarily functions during the S and G2 phases [26]. HRR involves a sequence of interconnected downstream elements that utilize DNA strand invasion and template-guided DNA repair synthesis. Through these processes, HRR ensures precise and accurate repair, thus preserving the integrity and fidelity of the genetic material [27]. The HRR process can be systematically divided into three sequential phases. First, the resection of the ends of DSBs occurs, which is a critical initial step that prepares the damaged DNA for subsequent repair. Next, the synthesis of the missing sequence takes place, relying on a template to accurately reconstruct the deleted portion of the DNA strand. Finally, the process culminates in the annealing and ligation stages, where the newly synthesized DNA segments are accurately aligned and covalently joined. In summary, HRR proceeds through three sequential steps: DSB end resection, template-directed sequence synthesis, and subsequent annealing and ligation, ensuring the faithful repair of damaged DNA [28]. HRR preferentially utilizes sister chromatids as templates over homologous chromosomes [29], the process necessitates strand invasion facilitated by the recombinase RAD51 [30].

The relationship between DNA damage and immunotherapy has garnered considerable attention in recent years, emerging as a critical area of study in oncology. DNA damage is crucial not only in initiating and advancing cancer but also in significantly influencing the effectiveness of immunotherapy. DNA damage plays a dual and crucial role, promoting harmful processes within the body while also influencing the effectiveness of treatments that leverage the immune system to fight disease. This dual nature underscores its essential impact on both the development of cancer and the formulation of therapeutic approaches. Investigations reveal that damage to DNA can boost the immunogenic potential of cancer cells, leading to enhanced immune system activity against such malignancies. For instance, the inhibition of DDR mechanisms may potentiate tumor cells' sensitivity to immune checkpoint inhibitors. Moreover, the elevated mutational burden resulting from DNA damage can generate a greater number of neoantigens, thereby enhancing the efficacy of immunotherapy [31]. Conversely, the accumulation and incomplete repair of DNA damage might also facilitate immune escape, potentially compromising the success of immunotherapy. Therefore, understanding the complex relationship between DNA damage and the immune system will aid in the creation of novel immunotherapy approaches, improve treatment effectiveness, and reduce side effects associated with therapy.

The diverse array of natural resources in China includes many compounds with antitumor potential. These substances are gaining prominence due to their selective cytotoxicity, which affects cancerous cells while sparing normal cellular functions. As a result, they are being explored as effective chemotherapeutic agents to curb tumor onset and advancement. With their lower toxicity levels compared to conventional treatments, natural products are emerging as viable candidates for both preventing and treating tumors, thereby attracting increasing focus within the scientific community [32–35]. The field of natural product studies has identified several compounds that possess the capacity to facilitate or engage in DDR [2, 36, 37]. Natural compounds can be classified primarily according to their chemical structures into various categories, such as terpenoids, carotenoids (like α-carotene and β-carotene), and phenolic derivatives. Phenolic derivatives consist of multiple subcategories, including phenolic acids, flavonoids, stilbenes (such as resveratrol), coumarins, and tannins. In addition, these compounds also encompass alkaloids and other nitrogen-containing substances, organosulfur compounds like isothiocyanates and indoles, as well as allyl sulfides. Flavonoids are subdivided into several types, such as chalcones (Isoliquiritigenin), lignans, flavonols (e.g., quercetin and kaempferol), flavanols (e.g., epigallocatechin), isoflavones, and anthocyanins [38]. Quercetin and lignans, which belong to the group of flavonoids, demonstrate remarkable antioxidant, anti-inflammatory, anticancer, and cardioprotective effects [39–41]. Quercetin helps prevent heart disease and cancer through its ability to neutralize free radicals and inhibit inflammatory responses [42, 43]. The distinguishing feature of lignans lies in their remarkable anti-inflammatory and neuroprotective properties [44, 45]. Berberine, a notable alkaloid, possesses antibacterial, blood glucose-lowering, and lipid-modulating activities. These characteristics have facilitated its broad use in managing infectious diseases, diabetes, and cardiovascular disorders [46]. Cantharidin possess strong anti-important effects and play a common role in cancer treatment regimens. On the other hand, apigenin is known for its antioxidant, anti-inflammatory, and anticancer attributes, which enhance its effectiveness in combating tumors and inflammation [47–49]. These naturally derived compounds are essential in preventing and treating various conditions, including cancer, inflammation, infections, and metabolic disorders, owing to their wide-ranging pharmacological effects. Numerous studies have demonstrated that specific natural products facilitate repair by interacting with key enzymes in the pathway [50]. A diverse array of natural products has been discovered to exert a substantial influence in the management of various ailments [51–53]. The present review will focus on the modulation of key enzymes in the DDR mechanism by various natural bioactive compounds and their implications in disease development.

## Natural compounds influence critical enzymes associated with BER-induced disease development

Bioactive compounds from natural origins can influence DDR by targeting essential enzymes in BER pathway. This pathway includes critical enzymes such as 8-oxoguanine DNA glycosylase 1 (OGG1), AP endonuclease 1 (APE1), DNA polymerase, DNA ligase, and nucleic acid endonuclease (NEIL1). These enzymes play pivotal roles in repairing DNA damage through the BER process [1]. Repair process-associated genetic defects give rise to malignancies, inflammatory conditions, senescence, and neurodegenerative disorders [54–56].

NEIL1 modulates glycolipid metabolism via mechanisms that are influenced by cellular redox status and mitochondrial function. Downregulation of this DNA repair enzyme is associated with increased genomic instability, impaired mitochondrial energy production, elevated circulating phospholipid and triglyceride levels, heightened liver inflammatory responses, and excessive insulin secretion [57]. The administration of berberine enhances insulin secretion, ameliorates insulin resistance, suppresses adipogenesis, mitigates adipose tissue fibrosis, alleviates hepatic steatosis and improves intestinal dysbiosis [58, 59]. Berberine shows considerable promise as a therapeutic agent for effectively managing metabolic disorders [60–62]. Further investigation, especially through clinical trials, is required to clarify its molecular mechanisms and targets.

NEIL1 coordinates glycolipid metabolic regulation through pathways influenced by oxidative stress responses and mitochondrial functional capacity. Repression of this glycosylase is associated with increased DNA damage accumulation, impaired mitochondrial bioenergetics, elevated serum lipid profiles (including phospholipids and triglycerides), hepatic inflammation, and disrupted insulin regulation [63]. The BER process induces genomic stress by elevating Endonuclease III-like protein 1 (NTH-1) levels, thereby promoting age-related neurodegeneration in the *Hidradenitis elegans* Parkinson's disease (PD) model [64]. Multiple bioactive constituents in traditional Chinese medicine (TCM) exhibit neuroprotective effects, potentially mediated through regulatory influences on the BER mechanism. Notably, certain qi-tonifying botanicals may enhance mitochondrial function and alleviate genomic instability, mirroring the protective outcomes observed under NTH-1 pathway inhibition. Moreover, the Poly pharmacological nature of TCM preparations, which simultaneously interact with multiple molecular targets, aligns well with the complex network of cellular signaling cascades examined in this study. This suggests that exploring the effects of TCM on the BER pathway and associated signaling pathways may elucidate potential mechanisms for PD treatment. Such research could provide valuable insights for the development of novel TCM-based drugs or therapies for PD, thereby advancing the role of TCM in neurodegenerative disease treatment. Berberine, an isoquinoline alkaloid obtained from traditional Chinese medicinal plants, shows promise as a treatment for neurodegenerative disorders. This potential is attributed to its ability to inhibit critical enzymes involved in these diseases, reduce intracellular oxidative stress and neuroinflammation, stimulate autophagy, and protect neurons from damage [65]. Moreover, studies in the literature have shown that natural compounds like ginsenoside, epigallocatechin gallate, soy isoflavones, curcumin, resveratrol, tanshinone, ligustilide, ginkgo biloba extract, and baicalein possess neuroprotective properties. These findings open avenues for new therapeutic approaches and offer significant insights into the creation of innovative drugs and methods for addressing neurodegenerative disorders [66]. NEIL3 acts as a DNA glycosylase in BER pathway and plays an essential role in effective DDR, particularly in cells that divide rapidly. Reduced expression of NEIL3 leads to neurological abnormalities characterized by two main pathological features: a decrease in the number of microglia in the striatum and an exaggerated response from neuronal progenitors during hypoxia–ischemia reperfusion events [67].

**Fig. 1:**
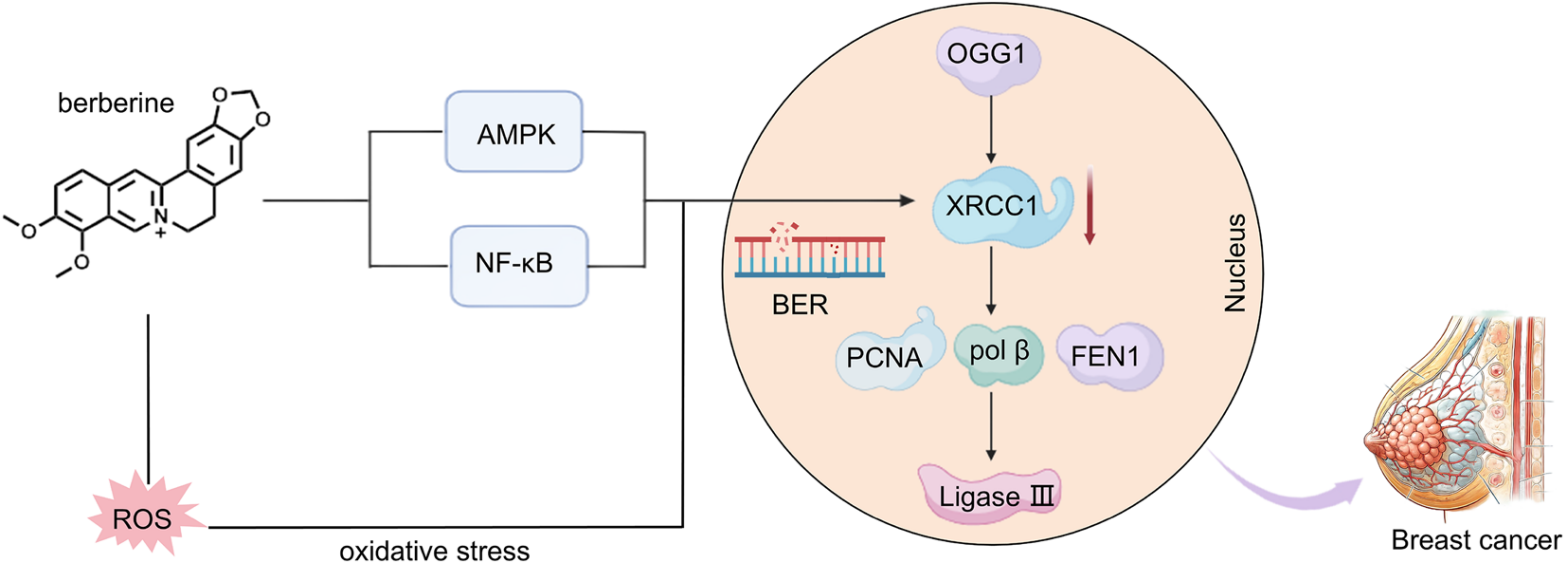
Mechanisms through which berberine treats breast cancer involve modulation of the XRCC1 enzyme in BER. Berberine can activate a cascade of transcription factors via AMPK signaling pathway, leading to upregulation of XRCC1 gene expression. Additionally, it facilitates the role of XRCC1 in the repair process by means of NF-κB signaling pathway. Furthermore, it indirectly enhances XRCC1 activity by mitigating oxidative stress-induced DNA damage

The induction of colorectal carcinoma by 1,2-dimethylhydrazine was attenuated by quercetin through the upregulation of key BER enzymes, including OGG1, APE1, and XRCC1. This enhancement in enzyme levels promoted more efficient DDR, resulting in a significant reduction in the formation of 8-oxo-dG [42]. XRCC1, a scaffolding protein that plays a crucial role in the BER pathway, in combination with etoposide and resveratrol, presents a promising therapeutic strategy for non-small cell lung cancer (NSCLC). This enhanced efficacy is attributed to resveratrol's ability to increase the chemosensitivity of etoposide by downregulating XRCC1 expression [68]. Berberine downregulates the expression of XRCC1, thereby disrupting the XRCC1-mediated BER pathway. This disruption not only compromises cellular DNA repair mechanisms but also enhances the sensitivity of triple-negative breast cancer (TNBC) cells to chemotherapeutic agents, potentially improving their responsiveness to treatment [69]. The mechanism by which berberine exerts its effects in the treatment of breast cancer (Fig. 1). Thujaplicins suppress the function of DNA polymerase β and λ within the X family, consequently impacting both BER and NHEJ pathways. This suppression increases the sensitivity of various cancer cells to bleomycin and temozolomide [70]. The application of lignocaine in lung squamous carcinoma cells resulted in an elevation of OGG1 levels [71].

## The role of natural products in neuroendocrine regulation and their potential applications in cancer and metabolic disorders

The NER process encompasses two distinct pathways: global genome repair (GGR) and transcription-coupled repair (TCR) [72–74]. The body undergoes TCR to address transcription-blocking DNA lesions (TBL) in genomic DNA [75]. The initiation of TBL induces transcriptional stress, disrupting the normal regulation of gene expression and leading to severe consequences such as carcinogenesis, accelerated aging, and neurodegenerative disorders [76, 77]. The key enzymes involved in the TCR pathway include Cockayne syndrome protein B (CSB), CSA, UVSSA, RNA polymerase II, and other essential components [78]. The GGR mechanism operates genome-wide, repairing NER-related damage at any genomic location and throughout all stages of the cell cycle [79]. GGR primarily involves two key complexes: CRL4-DDB2 and Xeroderma pigmentosum complementation group C (XPC). The process is initiated by the assembly of the TFIIH complex, together with XPA, Replication protein A (RPA), and the endonucleases XPG and ERCC1-XPF [80].

Excision repair cross-complementation group 1 (ERCC1) plays a crucial role in regulating pancreatic β-cell activity and insulin responsiveness, and its association with the onset of diabetes and its related complications has been well established. In a mouse model with adipose tissue-specific ERCC1 knockout, elevated DNA damage levels triggered a significant increase in inflammatory cytokines, such as interleukin-6 and tumor necrosis factor, within the adipose tissue. Consequently, this inflammatory response contributed to the development of impaired glucose tolerance in the mice [81]. From the perspective of TCM, numerous Chinese herbs possess anti-inflammatory and metabolism-regulating properties. For instance, astragalus and lycium barbarum may enhance the regulation of fat metabolism by modulating DDR mechanisms or inflammatory pathways. Future studies should investigate the effects of TCM on these metabolic pathways, which may pave the way for developing novel therapeutic strategies for disorders associated with fat metabolism. While there is no direct evidence linking natural products that can target ERCC1 for diabetes treatment, certain natural products have demonstrated potential in this area. For example, polysaccharides extracted from mulberry leaves demonstrate a range of bioactive properties, such as lipid-lowering, blood glucose reduction, antioxidant, and anti-inflammatory effects. They are also capable of protecting pancreatic islet cells, alleviating insulin resistance and regulating intestinal flora [82]. However, the pharmacological mechanism of mulberry leaf polysaccharides in diabetes treatment remains incompletely elucidated. Curcumin, a bioactive compound derived from turmeric, demonstrates a wide range of physiological and pharmacological benefits. These include potent antioxidant and anti-inflammatory properties, as well as anticancer, neuroprotective, and antidiabetic effects, highlighting its potential for diverse therapeutic applications [83, 84]. A substantial body of research has consistently demonstrated curcumin's efficacy in both the prevention and management of diabetes [85]. The naturally derived compound Rhizoma Coptidis has shown significant potential in addressing a variety of conditions, including tumors, metabolic disorders, and inflammatory diseases. Its therapeutic properties suggest it could play a crucial role in overcoming diverse health challenges, offering a promising avenue for advancements in medical research and treatment [86, 87]. Despite the therapeutic potential of these natural products, their specific pharmacological mechanisms of action remain elusive and necessitate further investigation by researchers.

A subset of individuals diagnosed with XP may progress to develop a severe and debilitating neurodegenerative condition known as XP neurological disease [88, 89]. This condition progressively affects the nervous system, leading to substantial neurological deterioration and significantly diminishing the quality of life for affected individuals. Mutations in the XPA, XPD, and XPG genes are identified as primary contributors to neurological impairment in XP patients. These genetic alterations are strongly associated with severe neurological deficits. Conversely, mutations in the XPC, XPE, and XPV genes generally do not exhibit a direct association with neurological abnormalities, suggesting that these genes play a lesser role in the progression of neurological issues in XP patients [90]. The application of polyphenols as a multi-targeted therapeutic strategy presents a promising and practical approach for addressing neurodegenerative disorders, which are often difficult to manage with conventional treatments like glutathione supplementation and cholinesterase inhibitors. By simultaneously influencing multiple pathways, polyphenols may offer a more comprehensive therapeutic approach that addresses the multifaceted nature of these diseases, potentially enhancing patient outcomes in ways that conventional drugs cannot achieve. This innovative strategy holds considerable promise for improving the efficacy of treatments targeting neurodegenerative diseases [91]. Magnoflorine exhibits considerable potential as a promising therapeutic candidate for addressing neurological disorders, particularly Alzheimer's disease (AD). Its distinctive pharmacological properties indicate that it may play a pivotal role in the management of these conditions, providing a novel therapeutic avenue and potentially enhancing outcomes for affected individuals [92, 93]. The potential of curcumin in the treatment of neurodegenerative diseases, including AD and PD, has been demonstrated [84, 94, 95].

**Fig. 2:**
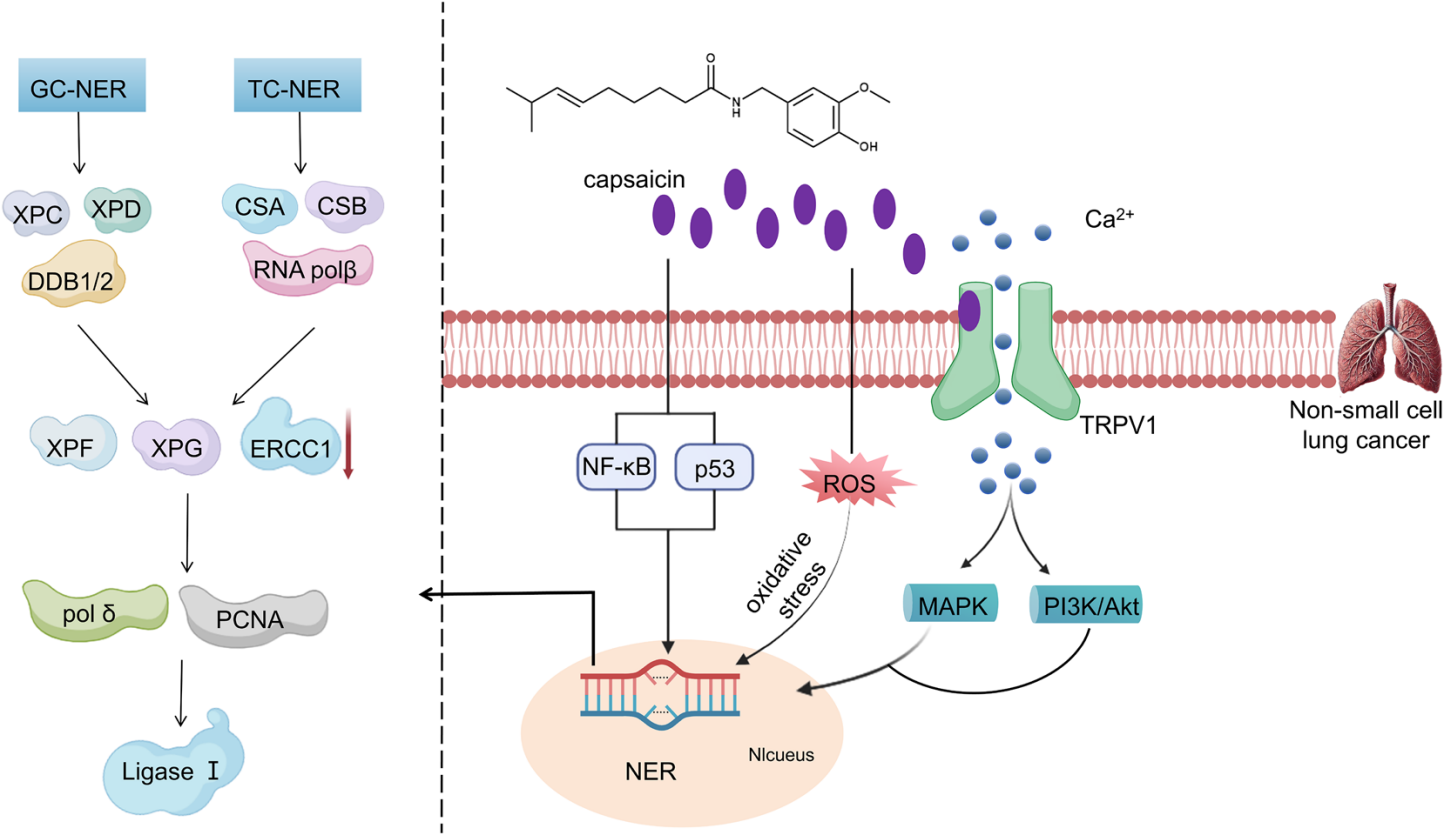
Mechanisms by which capsaicin treat NSCLC involve modulation of the ERCC1 enzyme in the NER pathway. Capsaicin effectively regulates ERCC1 expression through its binding to the transient receptor potential vanilloid subfamily 1 receptor, thereby initiating downstream signaling pathways such as MAPK and PI3K/Akt, ultimately leading to an increase in intracellular calcium ion concentration. Additionally, capsaicin inhibits ERCC1 expression by activating transcription factors including NF-κB and p53. Furthermore, capsaicin induces oxidative stress resulting in the generation of substantial amounts of free radicals and ROS, consequently causing DNA damage within cancer carcinoma

Capsaicin has shown significant potential in enhancing the cytotoxic impact of erlotinib and effectively inhibiting cell proliferation in NSCLC cells. When used in combination, this therapy leads to a notable decrease in ERCC1 expression and a strong suppression of the AKT signaling pathway in both A549 and H1975 cell lines. These results indicate that capsaicin could be vital in increasing NSCLC cells' sensitivity to erlotinib, thereby potentially improving the effectiveness of erlotinib in treating this aggressive type of cancer [96]. The therapeutic effects of capsaicin on NSCLC are mediated through its modulation of the ERCC1 enzyme, a key component of the NER pathway. By influencing this critical enzyme, capsaicin significantly alters cellular repair processes, thereby enhancing its efficacy in combating the disease (Fig. 2). Retigeric acid B enhances the efficacy of cisplatin in hormone-resistant prostate cancer cells by modulating the NER pathway. Specifically, it targets key proteins including ERCC1, TFB5, and RPA1, thereby potentiating the therapeutic effect of cisplatin. Additionally, Retigeric acid B may influence the MMR system by potentially interacting with the DNA Mismatch Repair Protein 2 (MSH2) and MSH6 proteins, which could contribute to enhanced therapeutic outcomes [97]. The concurrent application of Tretinoin and oxaliplatin in pancreatic cancer cell lines demonstrates a synergistic effect by downregulating key enzymes involved in the NER process. Specifically, this downregulation affects critical enzymes such as XPA, XPB, XPC, ERCC1, XPD, and XPF, which are essential for DNA repair mechanisms. However, in contrast to breast cancer cells where an elevation in γH2AX levels is observed along with the presence of DNA DSBs [98].

Genetic mutations in NER-associated genes can lead to various inherited disorders, such as XP, CS, and photosensitive trichothiodystrophy (TTD) [99–101]. XP, an exceptionally rare autosomal recessive disorder, is characterized by an extreme and often debilitating photosensitivity of the skin to ultraviolet radiation, making even minimal sunlight exposure a significant health risk [102, 103]. Genetic mutations in XPA, XPB (ERCC3), XPC, XPD (ERCC2), XPE (DDB2), XPF (ERCC4), and XPG (ERCC5) are commonly found in individuals with XP. These defects lead to compromised NER mechanisms, which are hallmark features of the disorder [100, 104]. The heightened vulnerability to DNA damage observed in XP patients stems from mutations in genes that play a crucial role in the NER pathway. This pathway is vital for repairing DNA lesions caused by Ultraviolet (UV) radiation [105]. The progression of skin cancer is a multifaceted and incremental process that initiates when the cellular systems tasked with repairing UV-induced DNA damage become impaired or cease to function effectively. Prolonged exposure to UV radiation triggers the formation of specific DNA abnormalities, including cyclobutane pyrimidine dimers (CPDs) and 6–4 pyrimidine-pyrimidone (6-4PPs) photoproducts, which play a pivotal role in this cancerous transformation. If these abnormalities are not adequately repaired, they tend to accumulate, leading to genetic mutations. As time progresses, the buildup of such unrepaired damage can interfere with normal cellular functions, escalating the risk of malignancy and the onset of skin cancer [106]. The presence of genistein in normal skin significantly reduces the formation of CPDs induced by UVB radiation, demonstrating its remarkable photoprotective efficacy [107]. The disorders of CS and TTD have been linked to deficiencies in NER, with mutations in the CS proteins specifically affecting only TCR [108]. Although patients with CS and TTD exhibit sensitivity to UV light, they do not manifest a predisposition to skin cancer. Mutations in the XPG gene can give rise to both XP and CS. XP arises from point mutations in the XPG gene that impair NEIL1 activity, while CS stems from truncating mutations in the XPG gene that disrupt transcriptional activity.

## The emerging role of natural products in tumor therapy: MMR enzymes and Lynch syndrome (LS)

The MMR contains two groups of key enzymes, namely the homologues of the bacterial MutLS system. The MutS family of enzymes comprises three key proteins: MSH2, MSH3, and MSH6. These proteins are essential for recognizing and binding mismatched DNA base pairs during the DNA repair process. On the other hand, the MutL family comprises four key proteins: MLH1, MLH3, Postmeiotic segregation increased 1 (PMS1), and PMS2. These proteins collectively function synergistically to facilitate the correction of DNA mismatches through interactions with MutS enzymes, thereby preserving genomic integrity and mitigating the risk of disease development, including malignancies [109].

LS results from heterozygous mutations in the germline of critical MMR genes, specifically MSH2, MSH6, MLH1, and PMS2. These genes play an essential role in correcting DNA replication errors, and mutations in these genes lead to defective repair mechanisms, thereby significantly increasing the risk of various cancers, particularly colorectal cancer [110]. LS can also be triggered by the deletion of the 3' end of the EPCAM gene. This genetic alteration disrupts the normal expression of MSH2, thereby contributing to the onset of LS [111]. The patients with tumors associated with LS encompass colorectal, endometrial, ovarian, gastric, small bowel, hepatic and biliary tract, urinary tract, and cutaneous malignancies [112]. Individuals harboring mutations in MSH6 and PMS2 exhibit a significantly elevated risk of developing breast cancer [113]. Most cases are characterized by somatic mutations, with around 20% linked to LS. In sporadic prostate cancers, microsatellite instability (MSI) is mainly associated with loss-of-function alterations in the MSH2 and MSH6 genes. These genes play a vital role in MMR pathway. Mutations in these genes compromise the ability to correct DNA replication errors, leading to an increased accumulation of MSI. On the other hand, the occurrence of MSI in colon and endometrial cancers is predominantly attributed to the epigenetic silencing of the MLH1 gene, primarily via DNA methylation. This silencing impairs the normal function of MLH1 within the MMR pathway, leading to genomic instability and facilitating tumorigenesis in these tissues [114]. Although the role of MSH6 in various tumors is under investigation, there is currently no widely recognized specific natural product that directly targets MSH6 for tumor treatment. Elemene, an effective sesquiterpene component extracted from Zingiberaceae, exhibits significant therapeutic effects on lung, breast, and pancreatic cancers by inducing apoptosis of tumor cells through inhibition of their DNA synthesis [115, 116]. Currently, numerous natural compounds, including curcumin, resveratrol, and soy isoflavones, are being investigated for their potential effects on tumor development, attributed to their anti-inflammatory properties [117–119]. The possibility of modulation of MSH6 expression is also being considered. The natural compounds mentioned here offer potential avenues for the treatment of LS-associated cancers by modulating cellular signaling pathways, influencing gene expression and repair mechanisms, among other mechanisms. The mechanism of LS is intricate and involves a multitude of genes and signaling pathways. Future studies should not only explore further into the functions of MSH6 and its associated genes but also assess the potential of natural products in treating LS-related cancers, particularly their impact on tumor cell DNA repair mechanisms. The studies will offer a more robust theoretical foundation and practical guidance for the implementation of precision medicine in cancer therapy.

## Enhancing understanding of NHEJ-related enzymes: their therapeutic potential in metabolic and neurodegenerative disorders

The key enzymes involved in NHEJ include Ku70/80 proteins, DNA-PKcs, LIG4, XRCC4, and other factors [17, 120, 121].

DNA-PK is a crucial participant in NHEJ and has also been implicated in various components of DDR [122–124], which plays a vital role in the transcriptional regulation of adipogenesis [125]. DNA damage activates DNA-PK, leading to disrupted energy metabolism in skeletal muscle. This metabolic disruption ultimately contributes to increased insulin resistance, thereby exacerbating metabolic dysfunction. In contrast, reduced DNA-PK activity has been shown to promote improved glucolipid metabolism. Mice subjected to a high-fat diet with diminished DNA-PK activity exhibit enhanced glucose tolerance and insulin sensitivity. Furthermore, these mice show a lower incidence of obesity and hyperglycemia, indicating a beneficial metabolic adaptation [126]. Targeting the inhibition of DNA-PK shows significant potential as a highly effective therapeutic strategy for combating obesity and managing type 2 diabetes. Modulating DNA-PK activity could provide novel approaches to enhance metabolic function and insulin sensitivity, thereby potentially slowing the progression of these chronic conditions [127].

Amyotrophic Lateral Sclerosis (ALS) is a severe neurological condition marked by the gradual deterioration of motor neurons, which include both those in the cerebral cortex and those in the spinal cord. This degenerative process affects both upper and lower motor neurons, leading to a debilitating disorder. This neurodegenerative process disrupts neural signaling pathways, leading to widespread muscular denervation and subsequent muscle atrophy. The disease is characterized by a progressive loss of voluntary motor control, culminating in severe physical disability as the neuromuscular system experiences irreversible degeneration [128]. The occurrence and advancement of ALS are linked to deficiencies in NHEJ [129]. AD is a progressive neurodegenerative disorder marked by the gradual decline in brain function, with significant impacts on cognitive abilities and memory processes. This neurodegenerative disorder predominantly affects the cerebral cortex and hippocampal regions, resulting in the accumulation of abnormal protein deposits and the subsequent disruption of synaptic connections between neurons. These pathological changes lead to substantial impairment of higher cortical functions, ultimately causing severe disability in affected individuals [130]. The pathological manifestations of AD primarily involve localized neuronal death, as well as the accumulation of neurogenic fiber tangles and senile plaques, which are known as neuronal and extracellular lesions, respectively. Several studies have suggested a potential correlation between AD development and deficiencies in NHEJ, although further research is required to validate this association [131].

**Fig. 3:**
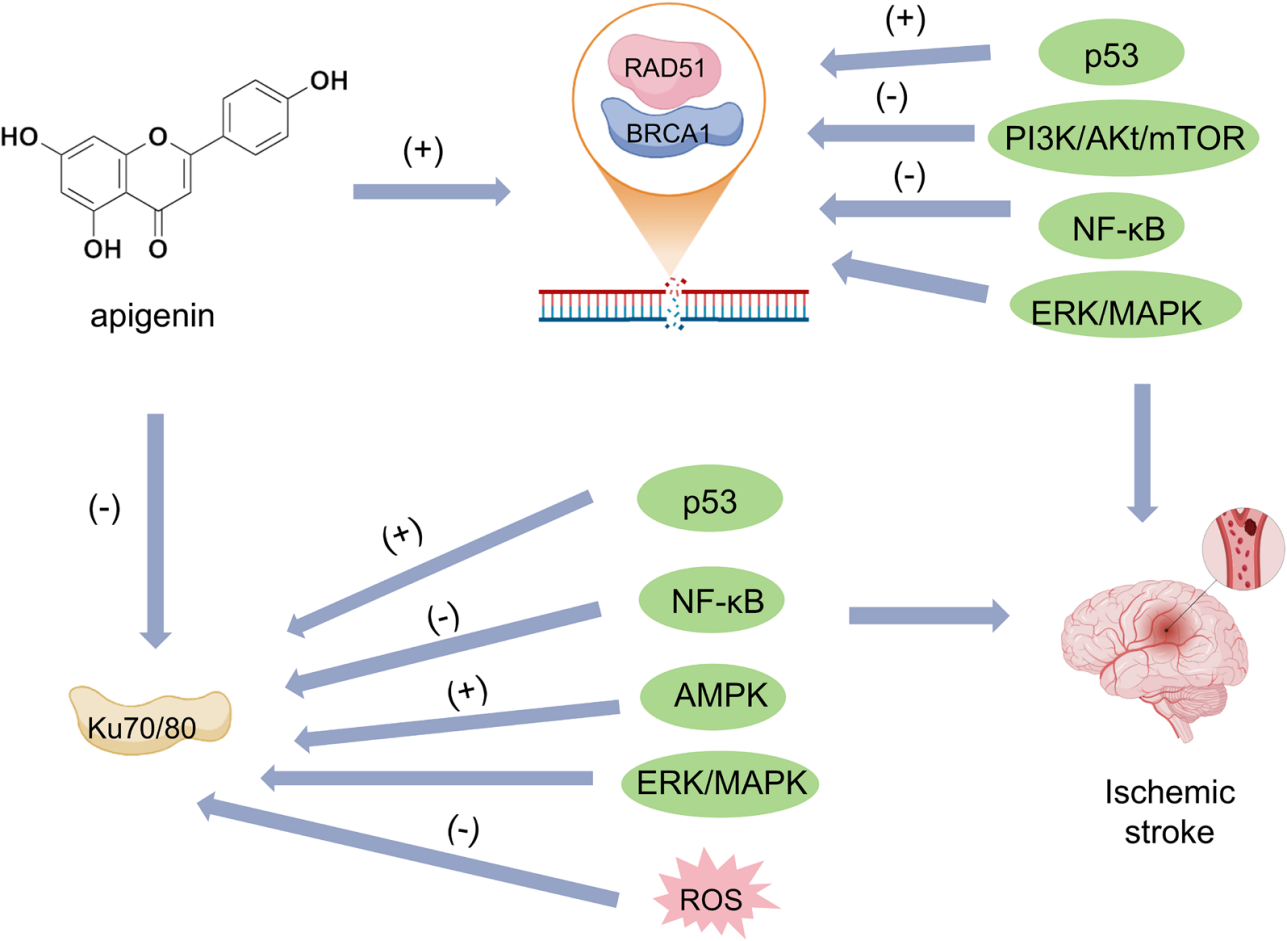
Apigenin holds promise in the treatment of ischemic stroke by modulating RAD51 and BRCA1 during HRR as well as Ku70/80 in NHEJ. It activates the p53 pathway, which may elevate the transcriptional activity of BRCA1, thereby indirectly regulating the expression of RAD51 and enhancing DNA repair processes. Additionally, apigenin upregulates the expression of Ku70/80 via the p53 pathway, further promoting DNA repair. It potentially inhibits cancer cell proliferation by suppressing the PI3K/Akt signaling pathway and decreasing cellular reliance on growth signals, thereby modulating the expression of BRCA1 and RAD51. Additionally, it exhibits anti-inflammatory properties, likely through the inhibition of NF-κB activity and the reduction of DNA damage induced by inflammation, thus safeguarding the functions of BRCA1, RAD51, and Ku70/80 and enhancing DNA repair mechanisms. It may modulate the expression and repair functions of BRCA1, RAD51, and Ku70/80 through the regulation of the ERK/MAPK signaling pathway. Additionally, it activates the AMPK pathway, thereby enhancing the expression of Ku70/80 and associated proteins, which contributes to increased DNA repair. Furthermore, it indirectly facilitates Ku70 expression by eliminating oxygen free radicals and mitigating oxidative DNA damage

Research investigations into redox-induced nucleic acid alterations within cerebral tissues following ischemic events have primarily focused on assessing the vulnerability of neural populations. This scientific inquiry highlights the molecular mechanisms responsible for free radical-mediated genomic instability in diverse cellular components of the central nervous system under hypoxic-ischemic conditions. Following cerebrovascular occlusion, there is a significant surge in reactive oxygen species (ROS) production, which induces molecular alterations within neural tissues, impacting both cortical regions and myelinated pathways. Genomic instability manifests rapidly post-occlusion, and while molecular repair mechanisms may mitigate these changes, their efficacy is time dependent. This cascade of biochemical events contributes to tissue damage across multiple CNS compartments, with the severity and reversibility of nucleic acid modifications varying over time [132]. Therefore, the concern regarding oxidative DNA damage and repair has emerged as a significant focus in stroke research. Apigenin has been documented in scientific literature for its potential therapeutic effects on ischemic stroke by downregulating the expression of the pivotal enzyme Ku70 involved in NHEJ [133] (Fig. 3).

Genistein exerts its inhibitory effects on HRR and NHEJ pathways in glioblastoma and sarcoma cells following carbon ion radiation by preventing the phosphorylation of key proteins Ku80 and DNA-PKcs, and by slowing down the assembly of RAD51 foci [134, 135]. The concurrent administration of genistein and AG1024 demonstrates a synergistic effect in enhancing radio sensitivity in prostate cancer cells. This synergy is primarily due to the collective downregulation of crucial proteins involved in DNA repair processes. More specifically, this treatment combination effectively suppresses the expression of RAD51, which plays a vital role in HRR, and Ku70, an indispensable factor in NHEJ. By targeting these key repair mechanisms, genistein and AG1024 together markedly compromise the cells' ability to respond to DNA damage from radiation, thus increasing their susceptibility to radiation therapy and potentially leading to better therapeutic outcomes [136]. The combination of ellagic acid and bevacizumab for anti-angiogenic therapy (also impacting DNA repair by reducing ERCC1 and XRCC1 expression) enhances tumor radiosensitivity [137, 138]. The elevation in ROS induced by β-carotene, coupled with the subsequent activation of caspase-3, may lead to a reduction in Ku protein levels in gastric cancer cells. This decrease in Ku proteins, which are critical for maintaining genomic stability, could impair the cell's ability to efficiently repair DNA damage. Consequently, the accumulation of cellular injuries can trigger apoptosis mechanisms, leading to programmed self-destruction of these malignant cells. The interplay between increased ROS levels and caspase-3 activation, triggered by β-carotene, offers a plausible explanation for how this compound may increase the sensitivity of gastric cancer cells to apoptosis [139].

## The role of RAD51 and its associated enzymes in HRR and their potential targets in cancer therapy

The RAD51 family of proteins is represented in every organism and is a key enzyme in HRR [140]. The other key enzymes involved in HRR include BRCA1, BRCA2, and PALB2, which are known to play crucial roles in both developmental abnormalities and oncogenesis [141].

PARP1 is a multifaceted protein that plays an essential role in detecting DNA strand breaks and coordinating their repair. Beyond its well-established functions in repairing DSBs and replication fork damage, PARP1 significantly contributes to maintaining cellular homeostasis by regulating metabolic processes. It influences mitochondrial function, which is critical for energy production, and modulates oxidative metabolism, thereby affecting cellular stress responses. In addition to its role in DNA repair, PARP1 also plays a significant part in aging-related diseases by regulating metabolism and managing oxidative stress, both of which are essential for maintaining cellular integrity and function over time. This extensive range of activities positions PARP1 as a key factor in genomic stability and age-associated health conditions [16]. Mice overexpressing PARP1 exhibit obesity and reduced glucose tolerance, making them a valuable mammalian model for studying inflammation [142]. From the perspective of TCM, certain Chinese herbs may modulate PARP-1-related pathways. For instance, ginseng and astragalus, known for their anti-inflammatory features, could potentially improve the metabolic and inflammatory conditions in hPARP-1 mice. Future research could explore their regulatory mechanisms on PARP-1, which may provide fresh perspectives on managing related conditions.

**Fig. 4:**
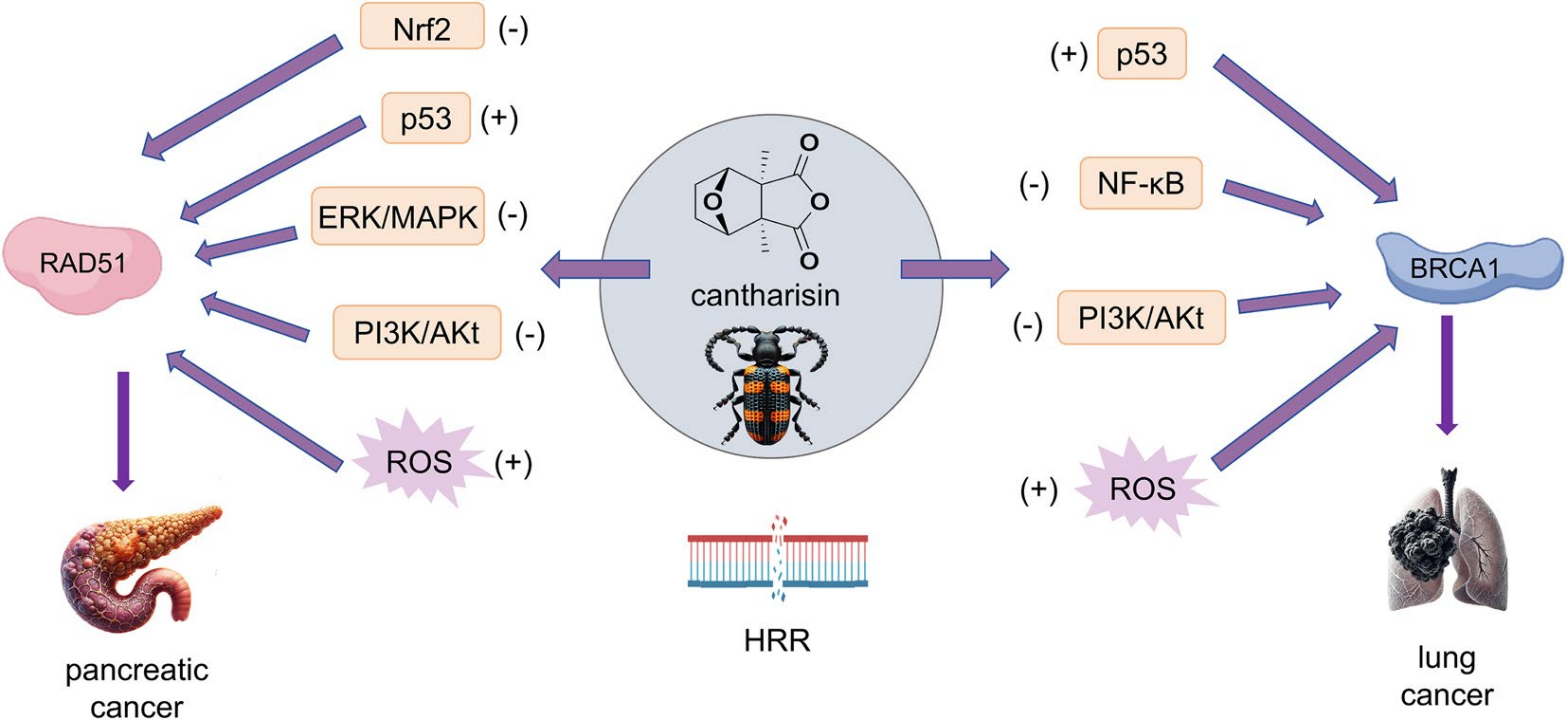
Cantharisin exhibits therapeutic potential against pancreatic and lung cancers by modulating RAD51 and BRCA1 in HRR pathway. By inhibiting the Nrf2 pathway, Cantharisin decreases the expression of RAD51, thereby increasing DNA damage in cancer cells. Additionally, it modulates the expression of RAD51 and BRCA1 via the activation of the p53 signaling pathway, which enhances DNA repair mechanisms and cell cycle checkpoints. It downregulates the expression of RAD51, thereby reducing the capacity of cancer cells to repair DNA by inhibiting the PI3K/Akt and ERK/MAPK signaling pathways. Additionally, it indirectly upregulates BRCA1 expression, thereby enhancing DNA repair mechanisms through the inhibition of the PI3K/Akt and NF-κB pathways. This compound contributes to the heightened reliance of cancer cells on DNA damage response by augmenting oxidative stress, which subsequently impairs the repair functions of RAD51 and BRCA1, leading to increased cellular apoptosis

**Fig. 5:**
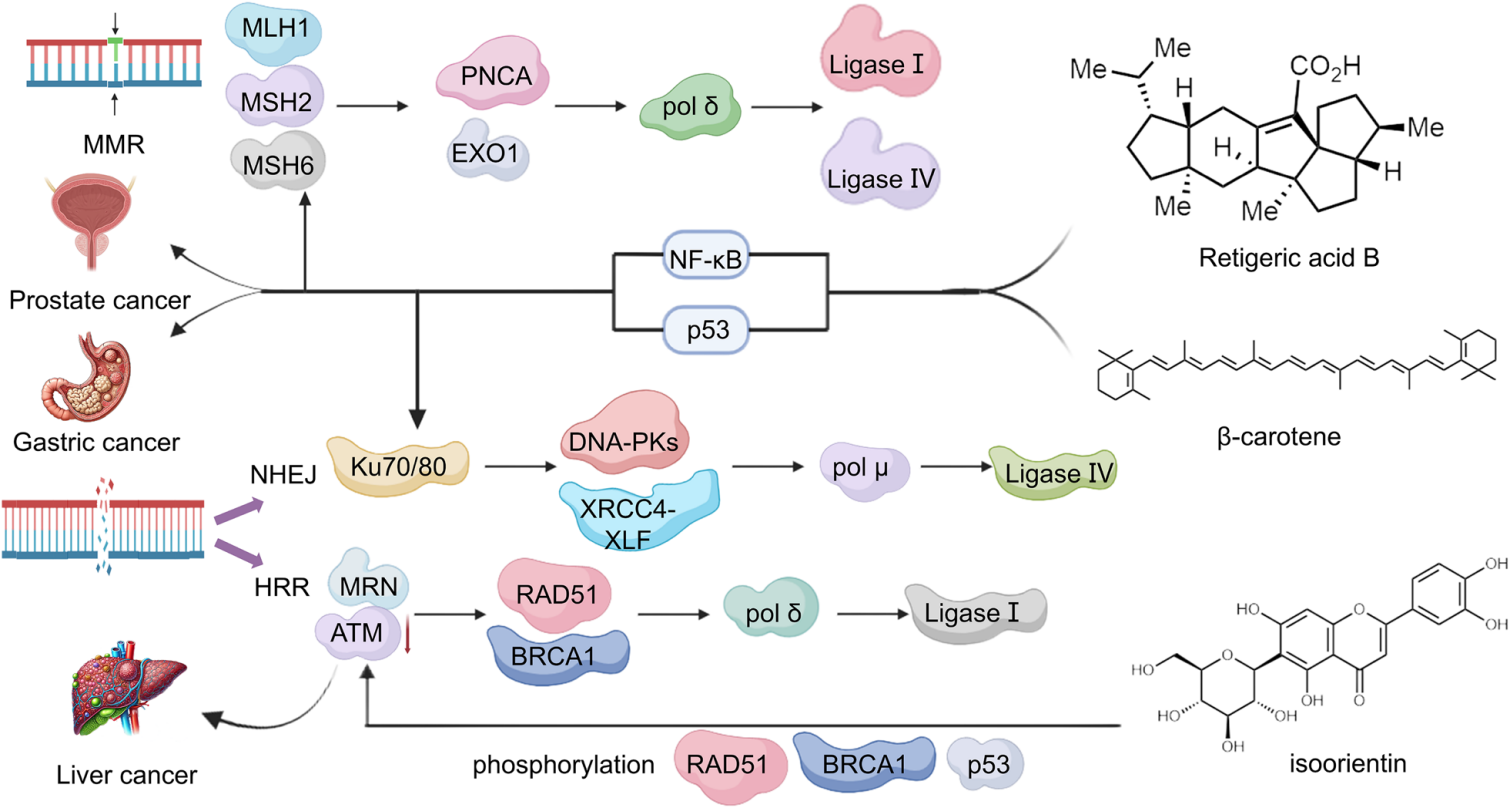
Retigeric acid B effectively treats prostate cancer by modulating the MSH2 and MSH6 enzymes involved in MMR. β-carotene exhibits therapeutic potential for gastric cancer by modulating Ku70 and Ku80 proteins in NHEJ. Isoorientin demonstrates efficacy against HCC by regulating ATM protein in HRR. Both retigeric acid B and β-carotene regulate the expression of relevant proteins through activation of transcription factors NF-κB and p53, respectively. ATM initiates the repair response by phosphorylating multiple target proteins including p53, BRCA1, and RAD51. On the other hand, Isoorientin inhibits the repair response by modulating ATM signaling pathway and reducing phosphorylation levels of ATM kinase, thereby impeding HCC intracellular DNA repair to achieve a therapeutic effect

Genetic alterations in genes that play a crucial role in HRR are often linked to the development of estrogen-dependent cancers, including breast cancer [143]. Berberine has been shown to enhance the radiosensitivity of human breast cancer cells via a multifactorial mechanism. Furthermore, berberine downregulates the expression of RAD51, a critical protein in the HRR pathway that is essential for repairing DNA DSBs. By down-regulating RAD51, berberine compromises the cells' capacity to effectively repair radiation-induced DNA damage, thereby enhancing their vulnerability to the detrimental effects of radiation therapy and potentially improving therapeutic outcomes [144]. The concurrent administration of berberine and PARP inhibitors resulted in a synergistic effect, inducing apoptosis and markedly suppressing tumor progression. Moreover, this study demonstrated that the combined treatment exerted a significant impact on ovarian cancer cells by inducing elevated levels of oxidative stress and DNA damage, ultimately enhancing their sensitivity to PARP inhibition [145]. The radiosensitivity conferred by RAD51 on esophageal carcinoma is effectively down-regulated by berberine [146]. Elevated expression of BRCA1 and reduced mammary carcinogenesis induced by 7,12-dimethylbenz[a]anthracene were observed in rats treated with genistein [147]. The expression of key enzymes, such as RAD50 and RAD51, is downregulated by cantharidin, leading to a sensitizing effect on pancreatic cancer cells [148]. The administration of cantharidin resulted in a significant reduction in the expression level of BRCA1 in NCI-H460 human lung carcinoma [149] (Fig. 4). Isoorientin suppressed HRR in hepatocellular carcinoma (HCC) cells while sparing normal cells, which is linked to reduced activation of ATM and inhibition of phosphorylated pATM binding to the MRE11-RAD50-NBS1 complex [150]. The mechanisms through which isoorientin exert its therapeutic effects on HCC by modulating ATM within HRR (Fig. 5). In traditional cancer treatments, a widely used approach to eliminate cancer cells involves blocking DDR pathways. Ferulic acid improves the effectiveness of PARP inhibitors in breast cancer therapy by reducing the formation of RAD51 foci, which is essential for HRR. Furthermore, it extends the period that DSBs stay unrepaired. The accumulation of these unrepaired DSBs over time increases cancer cell sensitivity to PARP inhibition, thereby enhancing the therapeutic efficacy of the treatment. By disrupting DNA repair mechanisms, ferulic acid compromises the cellular ability to maintain genomic integrity, thus increasing cellular vulnerability to the effects induced by PARP inhibitors [151]. The inhibition of HRR is achieved by β-thujaplicin through downregulation of the key enzyme RAD51, thereby enhancing the susceptibility of osteosarcoma cells to ionizing radiation-induced damage [152]. Tretinoin reduces the protein expression of PARP1, XRCC1, and RAD51 in TNBC cells, consequently impairing the SSBR, BER and HRR pathways [153].

## Enhancing DNA damage tolerance and targeting TLS polymerases in cancer therapy

A hallmark feature of the DDR network is its ability to detect and rectify DNA damage, as well as structural challenges that occur during DNA replication. This critical function, referred to as DNA damage tolerance (DDT), allows the cell to preserve genomic stability in the face of such damage. DDT enables cells to continue DNA synthesis even when confronted with lesions or obstacles in the DNA template, ensuring that replication can proceed uninterrupted, and the integrity of the genome is maintained. This adaptive response is essential for cell survival, especially in scenarios where DNA repair mechanisms may be temporarily overwhelmed or unable to fully resolve the damage [154]. TLS is one of the modes utilized of DDT [155]. The synthesis of TLS necessitates the utilization of designated DNA polymerases [156, 157]. The majority of these polymerases are classified as members of the Y-family [158]. The common polymerases found in *E. coli* cells are polymerase IV and polymerase V [159] as well as Pols η, ι, κ, and Rev1 [160, 161] in the mammalian cells. The B-family DNA polymerase ζ plays an indispensable role in eukaryotic TLS, underscoring its critical importance in this process [3, 162, 163]. The presence of genetic defects associated with DDT has been correlated with the development of various disorders, characterized by symptoms such as increased susceptibility to cancer, neurological impairments, abnormalities in stem cell function, and premature aging [154].

Research has demonstrated that diverse TLS enzymes significantly contribute to the development of platinum-based drug resistance in cancer cells. Polymerase η plays a important role in error-free trans-damage synthesis through various cisplatin adducts [164]. The application of interstrand crosslink inducers in cancer therapy leads to a significant upregulation of Pol η expression in ex vivo conditions [165]. The down-regulation of Rev1 expression in ovarian cancer cells also leads to a decrease in cisplatin-induced mutagenesis and drug resistance [166, 167]. Genetic ablation of Rev enzymes in B-cell malignancy experimental systems markedly reduced tumor tolerance to platinum agents, as demonstrated by enhanced cytotoxic responses in both cellular assays and animal models [168]. Demonstrated a significant prognostic association with reduced overall survival rates in patients diagnosed with NSCLC following cisplatin or carboplatin therapies [169]. Dual suppression of both Pol η and ATR enhances cisplatin's cytotoxicity in refractory NSCLC by compromising DNA damage response pathways [170]. The larger active site of individual TLS DNA polymerases makes them attractive targets for anticancer therapy, as they can be specifically targeted unlike replicative polymerases. Screening for inhibitors of Pol κ, a TLS polymerase, has identified promising lead compounds that require further development [171]. In the realm of natural products, a multitude of compounds derived from nature offer innovative approaches for cancer therapy. For instance, paclitaxel, a compound extracted from the bark of the Pacific yew tree (*Taxus brevifolia*), promotes microtubule polymerization, inhibits their depolymerization, and disrupts the mitotic process in cancer cells. This compound has demonstrated efficacy in treating various cancers, including ovarian, breast, and lung cancer, thereby playing a pivotal role in oncological treatments [172]. Camptothecin, an alkaloid extracted from the Chinese dove tree (*Camptotheca acuminata*), functions primarily to inhibit topoisomerase I, thereby disrupting normal DNA replication and transcription processes, ultimately leading to apoptosis in cancer cells. Derivatives such as irinotecan and topotecan, developed based on camptothecin, have been extensively utilized in clinical settings, offering significant therapeutic benefits for patients with colorectal and small cell lung cancers [173]. Curcumin, the principal bioactive compound in turmeric, exhibits anti-inflammatory, antioxidant, and anticancer traits. It modulates cell signaling pathways and induces apoptosis, thereby influencing the biological behavior of cancer cells. Research has demonstrated that curcumin may regulate the DDR signaling pathway, thereby enhancing the sensitivity of cancer cells to cisplatin and reversing cisplatin resistance [174]. Further investigation into DDT's potential is essential to develop effective anticancer strategies and uncover tumor-specific vulnerabilities, thereby optimizing therapeutic outcomes. In the future, personalized cancer drugs targeting specific vulnerabilities in tumors will be achieved through the utilization of abundant natural products. Finally, a table was utilized to summarize the regulation of key enzymes involved in DDR mechanisms and their associated diseases (Table 1).

**Table 1:** The role of natural products in the mechanisms of DDR and their associated diseases

Category of natural product	Name of natural product	DDR mechanism	Regulates the activity of key enzymes	Related diseases	References
Flavonoid	Quercetin	BER	Promoting OGG1	Colorectal cancer	[42, 175]
	Luteolin	BER	Promoting OGG1	Squamous cell carcinoma of the lungs	[71]
	Quercetin	HRR	Promoting RAD51	Oncological disease	[176]
	Genistein	HRR, NHEJ	Inhibition RAD51, Ku70	Prostate cancer	[134]
	Genistein	HRR	Promoting BRCA1	Breast cancer	[147]
	Apigenin	HRR	Promoting BRCA1, RAD51	Ischemic stroke	[177]
	Apigenin	NHEJ	Inhibition Ku70	Ischemic stroke	[177]
	Quercetin	NHEJ	Regulate Ku70/80, DNA-PKcs	NSCLC	[178]
	Quercetin	NHEJ, HRR	Regulate DNA-PK, ATM	Leukemia	[179]
Lactone	Triptolide	BER, NER	Regulate ERCC1, PARP1 et al	Osteosarcoma	[180]
	Triptolide	NER	Regulate XPA, XPB, XPC, ERCC1, XPD, XPF	Pancreatic	[98]
	Triptolide	NER	Regulate PARP	Advanced stage melanoma	[181]
	Triptolide	NHEJ	Regulate DNA-PKcs, Ku80	Oncological disease	[182, 183]
	Triptolide	HRR	Inhibition ATM	Breast cancer	[153]
Alkaloid	Berberine	BER	Inhibition XRCC1	Triple negative breast cancer	[69]
	Capsaicin	NER	Inhibition ERCC1	NSCLC	[96]
	Piperine	NHEJ	Regulate DNA-PKcs, Ku70/80	Breast cancer	[184]
	Berberine	HRR	Inhibition RAD51	Breast cancer	[144]
	Berberine	HRR	Inhibition RAD51	Esophageal cancer	[146]
	Berberine	HRR	Inhibition RAD51	Ovarian cancer	[145]
Polyphenol	Resveratrol	BER	Promoting OGG1 and XRCC1	Diseases caused by alcoholism	[185]
	Resveratrol	BER	Inhibition XRCC1	NSCLC	[186]
	Resveratrol	NER	Possible regulation OGG1, XRCC1	XP, skin cancer	[186]
	Resveratrol	HRR, NHEJ	Inhibition ATM/ATR-P53 and Nbs1	Oncological disease	[186]
Other	Retigeric acid B	NER, MMR	Regulate ERCC1, MSH2, MSH6	Prostate cancer	[187]
	β-carotene	NHEJ	Regulate Ku70/80	Stomach cancer	[139, 188, 189]
	Cantharisin	HRR	Regulate RAD50, RAD51	Pancreatic	[148]
	Cantharisin	HRR	Regulate BRCA1	Lung cancer	[149]
	Isoorientin	HRR	Regulate ATM	Liver cancer	[150]
	Ferulic acid	HRR	Inhibition RAD51	Breast cancer	[151]
	β-Thujaplicin	HRR	Inhibition RAD51	Osteosarcoma cell	[152]

## Discussion

The present study provides a comprehensive review on the role of natural active ingredients with diverse structural characteristics in modulating the activity or expression of pivotal enzymes and investigates their correlation with disease development. By investigating the regulatory impacts of these active compounds on DDR, we can better understand their potential roles in managing diseases. Research indicates that natural bioactive components hold promise in decreasing DNA damage and improving repair efficacy through the enhancement of DNA repair processes. This showcases their broad applicability in treating a variety of conditions, including cancer and neurodegenerative diseases. This study elucidates the therapeutic mechanisms by which natural bioactive compounds modulate critical enzymatic components within DNA damage response systems, specifically DNA polymerases and repair-related proteins, through functional and expression modifications. Targeted modulation of these catalytic mediators shows significant potential in inhibiting pathological processes, particularly malignant proliferation and neurocognitive decline associated with degenerative disorders. The Poly pharmacological properties of natural bioactive agents enhance the precision and efficiency of repair processes. Tretinoin exemplifies a paradigm-shifting capacity within genomic maintenance frameworks, as its lactone moiety modulates enzymatic activities across four key DNA repair pathways: BER, NER, NHEJ and HRR. These interconnected regulatory mechanisms provide critical insights into the molecular drivers of oncogenesis and lay the foundation for innovative therapeutic approaches in oncology. Quercetin exhibits a dual regulatory role in disease development. As a biologically active flavonoid, quercetin modulates DNA repair mechanisms through two distinct pathways. Specifically, it influences the BER system by regulating OGG1 and alters HRR by targeting RAD51 and BRCA1. These findings underscore its pathophysiological significance in associated disorders and highlight potential therapeutic strategies that leverage quercetin's activities. Additionally, berberine demonstrates complementary regulatory functions alongside its anti-suit properties. Berberine, an alkaloid compound, not only regulates XRCC1 in BER pathway but also plays a crucial role in NER. This dual-function mechanism suggests that berberine has the potential to be developed into a new therapeutic approach for TNBC and NSCLC, highlighting its innovative significance in cancer treatment.

BER is strongly associated with a variety of diseases, particularly those involving SSBs. NEIL1 plays a prominent role in glycolipid metabolism, and its decreased expression leads to DNA damage and impaired mitochondrial function. This impairment is closely linked to the development of metabolic conditions, including diabetes. Natural compounds, such as berberine, demonstrate potential in managing metabolic disorders by improving insulin sensitivity and reducing liver inflammation. In the context of neurological disorders, NTH-1 upregulation has been linked to neurodegenerative changes observed in PD models. Natural substances such as berberine may provide neuroprotection by mitigating oxidative damage and inflammation. Defects in NER are associated with genetic disorders like XP and CS, resulting in increased sensitivity to UV light and a heightened risk of skin cancer. The involvement of ERCC1 in pancreatic β-cell function suggests that deficiencies in NER may also impact the development of diabetes. Natural compounds such as curcumin and mulberry leaf polysaccharides exhibit certain antidiabetic properties, although the precise mechanism behind this effect requires further comprehensive investigation. In neoplastic disorders, deficiencies in MMR are closely linked to LS, resulting in an elevated susceptibility to various types of cancer. Current research indicates that specific natural compounds can modulate tumor growth by regulating the activity of critical repair enzymes. For instance, the efficacy of natural products in cancer therapy is exemplified by their effectiveness against lung and breast cancers. Moreover, compounds like curcumin and resveratrol possess antioxidant and anti-inflammatory properties that can indirectly impact tumorigenesis. In general, modulation of DNA repair mechanisms through natural products holds promise as a therapeutic approach for metabolic, neurological, and oncological diseases. Future research should focus on uncovering the molecular mechanisms behind these compounds and carry out clinical trials to confirm their effectiveness.

The emerging body of research suggests significant associations between NHEJ and HRR in DNA DSBs and a range of metabolic, neurological, cardiovascular, and oncological disorders. Key enzymes involved in NHEJ, such as DNA-PKcs, Ku proteins, LIG4, and XRCC4, not only contribute to DDR but also participate in physiological processes including adipogenesis, insulin sensitivity, and energy metabolism. It was observed that mice with impaired DNA-PK activity demonstrated enhanced glucose tolerance on a high-fat diet, implying the potential of targeting DNA-PK as a therapeutic intervention for metabolic disorders. In the context of neurological diseases, aberrant NHEJ mechanisms have been closely linked to the onset of ALS and AD, suggesting that dysregulation of DNA repair pathways may play a pivotal role in neurodegenerative pathologies. The dysregulation in question may expedite the progression of diseases by facilitating oxidative damage and neuronal cell death. In terms of cardiovascular disease, investigations into ischemic stroke have demonstrated the critical role played by oxidative DDR mechanisms in the pathological process. Relevant studies have demonstrated that the utilization of compounds, such as apigenin, for enhancing the functionality of NHEJ and HRR may offer novel therapeutic strategies for ischemic stroke treatment. In cancerous conditions, disruptions in NHEJ and HRR are associated with tumor initiation and progression. Compounds such as berberine and PARP inhibitors, which specifically target DNA repair pathways, have demonstrated the ability to enhance the treatment sensitivity of cancer cells. This evidence suggests a promising strategy for advancing oncology therapeutics. Dysregulation in DNA repair systems not only disrupts cellular damage response but is also intricately associated with the initiation and progression of various pathologies, underscoring the therapeutic potential of modulating these pathways. These findings open new avenues for innovative research and highlight the critical need for comprehensive studies on these molecular interactions to enhance therapeutic outcomes and disease management.

By introducing DNA lesions using cytotoxic drugs, the TLS pathway is triggered, playing a vital role in repairing genetic material. While TLS helps preserve the integrity of the genome by enabling continued DNA synthesis past damaged sites, it may also enable cancer cells to withstand chemotherapeutic treatments, potentially leading to treatment resistance. Experimental analyses have demonstrated that Y-family TLS enzymes, notably Pol η and Rev1, serve as crucial mediators in chemoresistance pathways against Pt-containing cytotoxins such as cisplatin in malignant progression models. Pol η bypasses cisplatin adducts by accurately synthesizing them, enabling tumor cells to survive chemotherapy-induced stress. Furthermore, mechanistic studies demonstrate that reduced Rev1 transcriptional activity correlates with diminished mutagenic effects of cisplatin-based treatments and increased chemosensitivity in epithelial ovarian carcinoma models. This suggests that the abundance and functionality of Y-family TLS enzymes are critical determinants of malignant cellular viability. Clinical data indicate that elevated levels of Pol η are associated with lower survival rates in NSCLC patients, underscoring the significant role of TLS in enhancing tumor resistance to chemotherapy. Consequently, developing inhibitors that specifically target TLS polymerases represent a highly promising strategy for cancer treatment. It is anticipated that the development of such targeted therapies will enhance tumor sensitivity to chemotherapy. Moreover, these therapeutic strategies are anticipated to provide new perspectives and methods for tailoring cancer treatment plans to meet the specific needs of individual patients. Essentially, TLS functions not only as a component of DDR but also plays a dual role in tumor therapy, presenting both opportunities and challenges. Future investigations should concentrate on effectively targeting TLS polymerase to achieve a balance between inhibiting tumor growth and reducing chemotherapy resistance. Gaining a more comprehensive insight into the connection between TLS and tumor-specific vulnerabilities might open new avenues for the creation of innovative cancer treatments.

The potential of natural active ingredients in regulating DDR mechanisms offers novel avenues for disease prevention and treatment. Through comprehensive investigation into the mechanism of action of these compounds, we anticipate uncovering their diverse roles in metabolic, neurological, and oncological disorders. The multi-targeted mechanism of action exhibited by natural substances, particularly in the context of cancer treatment, may offer novel insights for developing personalized therapeutic strategies. Moreover, the potential of targeted therapies that specifically inhibit TLS polymerases to enhance chemosensitivity and overcome drug resistance should be further explored through rigorous validation in clinical trials. An in-depth exploration of the complex relationship between DNA repair processes and the onset of diseases will provide a robust basis for the creation of innovative and efficient treatment approaches. In conclusion, the utilization of natural active ingredients in modulating DDR exhibits promising potential, and forthcoming investigations will furnish us with a more profound comprehension, foster novel drug advancements, and ultimately enhance disease prevention and treatment.

## Data Availability

The datasets created and examined in this study can be obtained from the corresponding author upon reasonable request.
